# Prevalent Pest Management Strategies for Grain Aphids: Opportunities and Challenges

**DOI:** 10.3389/fpls.2021.790919

**Published:** 2022-01-10

**Authors:** Kun Luo, Huiyan Zhao, Xiukang Wang, Zhensheng Kang

**Affiliations:** ^1^State Key Laboratory of Crop Stress Biology for Arid Areas, College of Plant Protection, Northwest A&F University, Yangling, China; ^2^Shaanxi Key Laboratory of Chinese Jujube, College of Life Science, Yan’an University, Yan’an, China

**Keywords:** wheat, ecological regulation, resistant cultivar, induced defenses, RNA interference

## Abstract

Cereal plants in natural ecological systems are often either sequentially or simultaneously attacked by different species of aphids, which significantly decreases the quality and quantity of harvested grain. The severity of the damage is potentially aggravated by microbes associated with the aphids or the coexistence of other fungal pathogens. Although chemical control and the use of cultivars with single-gene-based antibiosis resistance could effectively suppress grain aphid populations, this method has accelerated the development of insecticide resistance and resulted in pest resurgence. Therefore, it is important that effective and environmentally friendly pest management measures to control the damage done by grain aphids to cereals in agricultural ecosystems be developed and promoted. In recent decades, extensive studies have typically focused on further understanding the relationship between crops and aphids, which has greatly contributed to the establishment of sustainable pest management approaches. This review discusses recent advances and challenges related to the control of grain aphids in agricultural production. Current knowledge and ongoing research show that the integration of the large-scale cultivation of aphid-resistant wheat cultivars with agricultural and/or other management practices will be the most prevalent and economically important management strategy for wheat aphid control.

## Introduction

Common wheat (*Triticum aestivum* L.) is the third most important staple food crop worldwide, and it is widely cultivated in more than 150 countries throughout the world, occupying approximately 220 million hectares worldwide and feeding approximately 4.5 billion of the world population (FAOSTAT: Food and Agriculture Organization of the United Nations, 2019). Under the scenario of a rapid increase in the human population and a decrease in the area of cropland worldwide, the major challenge for current wheat grain production is reaching a steady annual increase of 2% (Crespo-Herrera et al., 2015). Moreover, wheat plants in agroecosystems are exposed to different pests that cause substantial damage to wheat and severely threaten global food safety. Among them, wheat aphids severely threaten wheat production worldwide; the English grain aphid *Sitobion avenae* Fabricius, the bird cherry-oat aphid *Rhopalosiphum padi* L., the greenbug *Schizaphis graminum* Rondani, and the Russian wheat aphid *Diuraphis noxia* Kurdjumov (Hemiptera: Aphididae), are the most destructive and most commonly occurring grain aphid species (Elbert et al., 2008; Liu et al., 2012; Crespo-Herrera et al., 2015). These aphids exhibit parthenogenesis and the typical features of R-strategists, which could significantly increase their populations in a short time. Their feeding behaviors involve ingestion of wheat phloem sugar at a high rate and transfer of most phloem sap from their bodily fluids into honeydew (Douglas, 2006), resulting in significant wheat grain yield and quality losses in many wheat production areas around the world (Rabbinge et al., 1981; Liu et al., 2012). In addition, grain aphids are a common vector of barley yellow dwarf virus (BYDV), which causes wheat yellow dwarf disease, one of the most destructive cereal diseases in Europe, Asia and Africa (Fiebig et al., 2004; Tanguy and Dedryver, 2009; Sadeghi et al., 2010). This viral disease further aggravates the problem in cereal crops by increasing the fecundity of grain aphids feeding on virus-infected plants (Fereres et al., 1989; Hu et al., 2013).

Currently, chemical control is still the most important measure to combat grain aphids in agricultural production as it can effectively suppress wheat aphid populations in a short time. Among these chemical insecticides, the neonicotinoid and pyrethroid insecticides are the main option for controlling grain aphids on the global market (Foster et al., 2014; Miao et al., 2014). The widespread and frequent use of neonicotinoid and pyrethroid insecticides in farming significantly stimulates grain aphids to develop insecticide resistance (Foster et al., 2014). The resistance of grain aphids to pesticides has caused a gradual resurgence of these pests. Thus, the damage caused by grain aphids has become a continuous problem in most wheat-producing regions of the world.

To guarantee food safety worldwide, it is imperative to find efficient pest management measures to control the damage from grain aphids. Moreover, over recent decades, genetic and biochemical information used for developing resistance to grain aphids has greatly contributed to a comprehensive way of developing more practical and environmentally friendly control of grain aphids. Therefore, current knowledge and ongoing research about strategies and approaches for sustainable grain aphid management will be synthesized and discussed in this review.

## Ecological Regulation of Grain Aphids

The growing desire for sustainable agriculture has prompted the need to develop more sustainable pest management approaches, such as ecological regulation. Ecological regulation generally refers to the use of agronomic-based management for mediating tripartite plant-pest-biological control agent interactions in agricultural ecosystems, which provides the most economic and environmentally friendly pest management measure (Zhou et al., 2012). Predators or parasitoids therefore play a dominant role in the ecological regulation of pest population growth. There are several biological control agents of grain aphids, including lady beetles (*Adalia bipunctata* L. and *Coccinella septempunctata* L.), green lacewings (*Chrysoperla carnea* Stephens), parasitic wasps (*Aphelinus abdominalis* Dalman and *Aphidius avenae* Haliday), marmalade hoverflies (*Episyrphus balteatus* De Geer), and trombidiid mites [*Allothrombium ovatum* Zhang & Xin (Acari: Trombidiidae)] (Ma et al., 2007; Vandenborre et al., 2011). However, in many cases, the number of predators or parasitoids present in agricultural ecosystems may be insufficient to provide economic management of pests on crops (Ma et al., 2007).

It was demonstrated that intercropping could change the environmental conditions, in a way that increases natural enemy activity, regulates pest population dynamics and minimizes crop damage (Ma et al., 2007; Vandenborre et al., 2011). Intercropping is a traditional agricultural technique of cultivating two or more crop species within the same field. In comparison with monocropping, intercropping could greatly contribute to increased crop production by effectively using environmental resources and suppressing pest outbreaks (Ma et al., 2007). Intercropping of wheat and alfalfa (*Medicago sativa* L.) provides the most practical and economical approach for controlling wheat aphids. For instance, wheat–alfalfa strip cropping significantly increased both the abundance of *A. ovatum* larvae and the parasitization rate of *S. avenae* compared to wheat monoculture (Ma et al., 2007). This could be explained by the fact that strip cropping provided a wetter, shadier soil surface microclimate that caused adult female mites to lay more egg pods and that the non-furrowed areas of the intercropped fields provided a more suitable habitat for mite overwintering (Brust et al., 1986; Zhang and Li, 1996; Ma et al., 2007). The intercropping of wheat and oilseed rape (*Brassica napus* L.) could improve the effective biological control of wheat aphids by increasing the species richness of natural enemies of *S. avenae*, including *E. balteatus* and *A. avenae*, which may control wheat aphid infestation during the early wheat filling stage (Wang et al., 2010). Wheat intercropping with pea (*Pisum sativum* L.) or mung bean (*Vigna radiata* L.) could also support these findings (Xie et al., 2012). This control is likely because the odor of non-hosts could attract a greater number of lady beetles and parasitic wasps to regulate the population dynamics of *S. avenae* colonization on wheat plants than could wheat monoculture. Moreover, intercropping could interfere with the host preference and locating abilities of aphids because the odor released from the non-host overlaps with the odor of the host. Experimental evidence in wheat intercropping with resistant wheat cultivars confirmed that intercropping could be an economic agricultural practice to reduce aphid populations (Zhou et al., 2009). Therefore, intercropping of wheat and other crops or vegetables could be an alternative measure to increase the populations of predators or parasitoids to control the population growth of grain aphids; however, it is challenged by the rapid increase in aphid populations, especially during the filling stage of wheat plants.

In addition, the practice on management of the aphids species of vegetable and crops relied heavily on entomopathogenic fungi, including *Beauveria bassiana*, *Metarhizium anisopliae*, and so on (Kim and Kim, 2008; Bayissa et al., 2017). This could be one of the cost-effective aphid management measures when aphid populations are low, similarly, it is challenged by the rapid increase in aphid populations as well. Moreover, increasing concern regarding the beneficial effects of soil microorganisms on plant growth and resistance to biotic stresses has led to the widespread use of beneficial microorganisms as biocontrol agents in agricultural practice. The genus *Trichoderma*, such as *Trichoderma harzianum* or *T. atroviride* strain P1, are biocontrol agents for the potato aphid (*Macrosiphum euphorbiae* Thomas). Tomato seeds soaked in a fresh spore suspension of either *T. harzianum* or *T. atroviride* strain P1 resulted in adverse effects on the development period and longevity of aphids by triggering plant resistance responses and/or the release of volatile organic compounds to attract the aphid parasitoid braconid *Aphidius ervi* Haliday (Coppola et al., 2017, 2019). Although few studies have reported that the genus *Trichoderma* of soil microorganisms could mediate the population of grain aphids, the above evidence provides important clues that the soaking cereal seeds in a spore suspension of *Trichoderma* could enhance the resistance of cereal seedlings to grain aphids.

## High-Voltage Electrostatic Field (HVEF)-Mediated Control Measures of Grain Aphids

Attempts to utilize artificial HVEFs for economical pest control have attracted increasing attention. Initially, direct exposure of seeds to HVEFs was utilized to improve the germination rate, and this practice continues to be used today. In general, crop seeds lose viability during storage, and the longer the storage period prior to cultivation, the greater the amount of reactive oxygen species (ROS) that accumulate (Wang et al., 2009). Directly exposing seeds to an HVEF could activate the antioxidative defense system by increasing antioxidant enzyme activities to increase the viability of seeds (Wang et al., 2009). Moreover, in a recent study, Luo K. et al. (2016) reported that the use of wheat seeds directly exposed to an HVEF could induce biological and physiological changes in the plants, which adversely affected the population growth of the grain aphid *S. avenae*.

In recent decades, large-scale electrical utilization, including long-distance electric power transmission, medical equipment, communication appliances, and so on, has seriously increased the intensity of electrostatic fields that are pervasively present in the environment. Herbivore insects are not only particularly sensitive to environmental alterations but also typically exhibit strong adaptation traits, such as short generation times, high reproductive rates, genetic plasticity, and small body sizes. Therefore, extensive studies have focused on characterizing the adverse effects and adaptive strategies of herbivorous insects to novel electric environments. With direct exposure of herbivorous insects to extreme static electric fields, multitudinous adverse effects can be induced, including chromosome aberrations, paralysis, increased mortality, abnormal propolization, reduced longevity, and possible impairment of colony growth (He et al., 2016). For instance, in our previous studies, when *S. avenae* was directly exposed to an HVEF with an intensity of 4 kV/cm for 20 min, the aphids experienced a significant increase in development time and a reduction in total longevity (He et al., 2016). Those studies have suggested that direct exposure of herbivorous insects to an HVEF is a possible alternative measure to prevent damage caused by these insects. However, the intensity of the current electrostatic environment could not pose serious adverse effects on insects, and establishing extreme static electric fields in agroecosystems would greatly increase production costs. In comparison, direct exposure of seeds to HVEFs is a more reasonable method in agricultural production. To better evaluate the possibility of HVEF exposure as a pest control measure, the direct exposure of seeds and newborn nymphs of *S. avenae* to a 4 kV/cm HVEF for 20 min significantly increased the superoxide dismutase activity but reduced the peroxidase and catalase activities, which indicates that the production of H_2_O_2_ exceeds the amount that antioxidant enzymes can gradually digest (Luo et al., 2019a). The extensive accumulation of H_2_O_2_ increases the oxidative stress and even cellular cytotoxicity and reduces the performance of the aphids. Therefore, direct exposure of seeds to HVEFs has the potential to play an important role in the development of alternative economic and environmentally friendly integrated pest management strategies for grain aphids.

## Plant Lectins as Defense Proteins Against Grain Aphids

Building aphid resistance into wheat plants is considered to be an ideal measure for combating aphids in agricultural production because it is less detrimental to the environment. Compared with cumbersome and time-consuming traditional breeding, adopting recombinant DNA technology to insert resistance into crops is a reliable and effective method to accelerate the breeding of cultivars with substantial insect resistance (Smith and Chuang, 2014). It was demonstrated that plant lectins have the potential to play an important role in the development of integrated pest management strategies (Michiels et al., 2010). Plant lectins are a specific group of proteins with at least one non-catalytic domain that can competitively bind specific carbohydrates, either simple monosaccharides or more complex glycans, resulting in inhibition of the assimilation of sugars in the gut of herbivores (Peumans and Damme, 1995). In addition, plant lectins are highly resistant to proteolysis and can bind to insect proteins, mainly in the gut, and as a consequence, they can be retained within the insect body (Michiels et al., 2010). The above findings suggest that plant lectins can cause adverse effects on the development or fecundity of insects. Thus, genetically modified wheat plants expressing plant lectins have become an important focus in wheat molecular breeding programs.

Snowdrop lectin (*Galanthus nivalis* agglutinin; GNA) is the first plant lectin gene successfully engineered into elite wheat cultivars to combat grain aphids in agricultural production (reviews by Michiels et al., 2010). Over the past few decades, considerable progress has been achieved in the genetic expression of GNA in different wheat cultivars through callus bombardment, which gives the plants a higher level of resistance against cereal aphids (Stoger et al., 1999; Hogervorst et al., 2009; reviews by Michiels et al., 2010; Vandenborre et al., 2011). For instance, introducing the GNA gene into the wheat cultivar Bobwhite was shown to exert severe entomotoxic effects on the development and survival of the grain aphid *S. avenae* (Stoger et al., 1999). In addition, the genetic modification of rice, maize, sugarcane, potato or tobacco plants to express GNA has successfully conferred resistance against different species of aphids (Wang et al., 2005; reviews by Vandenborre et al., 2011).

Although feeding lectins to insects via transgenic plants seems to be a relatively natural system, the potential risk of exposing larvae of different aphid predators, such as lady beetles (*A. bipunctata* and *C. septempunctata*) and green lacewings (*C. carnea*), to GNA has been explored for a long time (Hogervorst et al., 2006). The transfer of entomotoxic effects of GNA along the food chain has potentially increased the intensity of exposure of predators or parasitoids to GNA. The novel environment induced by GNA exposure significantly reduced the fecundity, egg viability and longevity of those aphid predators/herbivores when either feeding on an artificial diet containing GNA or preying on aphids reared on GNA-producing transgenic plants (reviews by Vandenborre et al., 2011). Moreover, feeding on grains or vegetables carrying entomotoxic lectins could trigger local and systemic allergic reactions in many species of mammals (reviews by Vandenborre et al., 2011). Taken together, these results suggest that prior to the development of genetically modified crop varieties expressing plant lectins, it will be necessary to fully understand the mechanism of toxicity of GNA and assess the potential risks of adverse GNA effects on predators and dietary uptake by animals or humans. Unfortunately, relatively few studies have investigated this issue, and the agricultural use of wheat germplasms genetically modified with plant lectins remains relatively rare.

## Breeding Pest-Resistant Wheat Cultivars for Grain Aphid Control

In natural agroecosystems, some wheat germplasms have coevolved a range of constitutive defenses to control the damage caused by aphid attackers. The identification of suitable genotypes with constitutive resistance to pests and the introduction of these genotypes into cultivars has resulted in reduced pesticide usage and lower production costs worldwide by controlling damage from pests (Christou et al., 2006). In the last few decades, vast efforts have focused on identifying aphid-resistant genotypes by adopting the terminology of Painter as well as subsequent revisions, and many accessions of common wheat and wheat relatives have been identified as resistant to grain aphids, providing abundant germplasm resources with durable and active resistance to breed wheat cultivars with substantial aphid resistance (Wang et al., 2015). Prior to developing new cultivars, screening suitable aphid-resistant traits from aphid-resistant germplasms would facilitate plant breeders in selecting cultivars with qualified aphid-resistant traits or preferred categories of aphid resistance. These resistance traits include morphological and structural features as well as the synthesis of chemical compounds.

**FIGURE 1:**
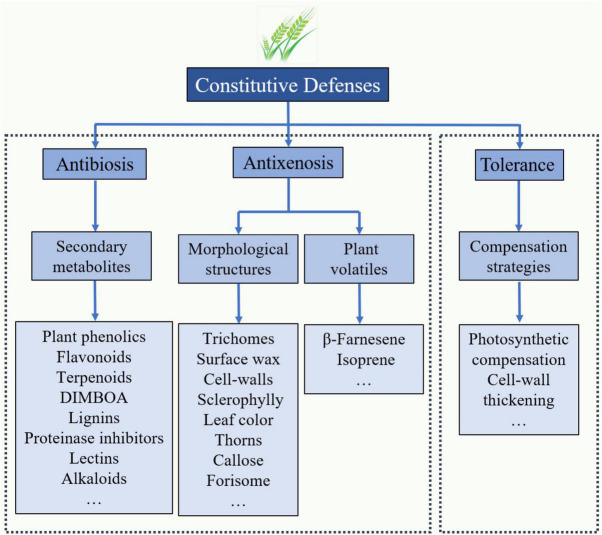
Proposed features and compounds associated with constitutive defense in response to grain aphids in resistant wheat lines. Most of the antibiosis and antixenosis traits exhibited in resistant lines are classified as qualitative traits (controlled by one or a few genes), while the tolerance traits are considered quantitative traits (controlled by numerous genes).

Three major commonly accepted categories exist for the insect resistance traits of plants: tolerance, antibiosis, and antixenosis (War et al., 2012). Among the types of resistance, tolerance is often a complex and polygenic trait that enables plants to compensate or withstand infestation from aphid damage and yield significantly more biomass than a susceptible plant under similar conditions (Figure 1). The evaluation of aphid tolerance always adopts the artificial aphid infestation method under field conditions (Hu et al., 2016; Luo et al., 2019b). During the pregenomics era, tolerance has been the preferred type of trait for conventional wheat breeding to obtain high-quality, high-yield, highly resistant cultivars without detrimental effects on human health (Inayatullah et al., 1990). In past decades, the molecular mechanisms of tolerance to many aphid species have been exploited in cultivars of alfalfa, barley, maize, rice, rye, sorghum and wheat (Smith and Chuang, 2014). For instance, wheat plants tolerant to the Russian wheat aphid, *D. noxia*, often exhibit increased photosynthetic rates, growth rates, stored root carbon and/or abilities to shunt stored carbon from roots to shoots (Kerchev et al., 2012; Smith and Chuang, 2014). The gene expression data in *D. noxia*-tolerant plants suggest that photosystem and chlorophyll genes involved in photosynthesis are highly expressed in the foliage of these plants. In a recent study, the results showed that winter wheat plants with higher tolerance to grain aphid infestation upregulated the relative expression of genes associated with photosystem I assembly protein and carbohydrate transfer and conversion several-fold (Luo et al., 2014, 2019b). During the grain-filling stage, large amounts of photoassimilates are transported into the endosperm, contributing to the grain yield, which compensates for the yield loss from the infestation of grain aphids.

Antibiosis is a type of resistance in which the plant produces allelochemicals or toxins, including plant phenolics, flavonoids, tannins, DIMBOA, and proteinase inhibitors, which significantly reduce herbivore growth and development (Figure 1). Antixenosis is a type of resistance in which certain characteristics of a plant, such as leaf surface wax, trichomes and cell walls, make it less attractive to herbivores (Figure 1; War et al., 2012). In many cases, the resistance of wheat germplasm to aphid feeding is classified into antibiosis resistance and/or antixenosis resistance; however, these effects are always difficult to separate in a single wheat germplasm because the traits associated with antibiosis and antixenosis resistance exhibit cooccurrence or coinheritance in the germplasm (Smith and Chuang, 2014).

To accelerate the process of breeding wheat cultivars with antibiosis resistance and/or antixenosis resistance to grain aphids, molecular marker technologies, such as simple sequence repeats (SSR), have been used in marker-assisted selection (MAS) to screen for aphid-resistant genes in wheat aphid-resistant lines (Bertin et al., 2004; Liu et al., 2012). When considering the closely linked loci of resistance genes, near-isogenic populations developed from crosses between aphid-resistant and aphid-susceptible parents have been successfully used to map and link the loci of aphid resistance genes to various types of molecular markers and develop chromosome maps of resistance genes (Smith and Chuang, 2014). In recent decades, over 10 *D. noxia*-resistant genes and 17 *S. graminum*-resistant genes were identified on wheat chromosomes by different molecular markers (Liu et al., 2001, 2005; Smith et al., 2004; Ricciardi et al., 2010). Most aphid resistance characterized in wheat is monogenic and inherited as a dominant trait. For example, the single dominant genes *Dn1*, *Dn2*, *Dn4*, *Dn5*, *Dn6*, *Dn7*, *Dn8*, *Dn9*, *Dn2412*, and *Dnx* were reported to confer resistance to Russian wheat aphids (Liu et al., 2001, 2005; Smith et al., 2004; Ricciardi et al., 2010); the candidate genes *Gb2, Gb3, Gb4, Gb5, Gb6, Gb7/Gbx1, Gb8, Gba, Gbb, Gbc, Gbd, Gbx, Gbx1, Gby, Gbz*, and *GbSkl* confer resistance to *S. graminum* (Boyko et al., 2004; Zhu et al., 2005; Aradottir and Crespo-Herrera, 2021). The recessive gene *dn3* from *Aegilops tauschii* (Coss.) has been linked to resistance to *D. noxia*, and the recessive gene *gb1* was the first identified resistance gene to greenbug and originated from *T. durum* (Miller et al., 2001; Dogimont et al., 2010). Recently, one of the *S. avenae* resistance genes *RA-1* was closely linked to the SSR molecular markers *Xwmc179*, *Xwmc553* and *Xwmc201* in the *T. durum* wheat line C273 (Liu et al., 2012). Our recent study revealed that the SSR molecular markers *Xgwm350* and *Xbarc70* are closely linked to an *S. avenae* resistance gene (*Sa2*) in the winter wheat genotype XN98-10-35 (Wang et al., 2015). Both SSR markers are monogenic and inherited as a dominant trait. Previous studies revealed that most of the characterized *D. noxia*, *S. graminum* or *S. avenae* resistance genes present in resistant cultivars have been located on wheat chromosome 7D based on evidence from molecular markers (Wang et al., 2015; Aradottir and Crespo-Herrera, 2021). It was reported that *Ae. tauschii* is the diploid progenitor of the D genome of common wheat and has carried a multitude of resistance genes, including those against wheat stripe rust, powdery mildew, wheat aphids, and so on (Zhu et al., 2005). In addition, these SSR markers will be valuable in MAS for accelerating the process of breeding wheat cultivars with resistance to grain aphids. Moreover, the candidate genes *Rdy2*, *Rdy3*, *Rdy4*, *Bdv1*, *Bdv2*, *Bdv3*, and *Bdv4* for resistance to BYDV have been identified by different molecular markers in barley and wheat cultivars or genotypes (Jarošová et al., 2016; Aradottir and Crespo-Herrera, 2021). However, few studies reported the identification or cloning of the dominant genes associated with *R. padi* resistance in wheat by adopting molecular markers, probably because of the polyphagy and wide host adaptation of *R. padi* (Crespo-Herrera et al., 2014). In addition, most of characterized *R. padi* resistance genes are controlled by quantitative trait loci (QTLs). For instance, Crespo-Herrera et al. (2014) reported three QTLs in the first report on the genetic mapping of *R. padi* resistance in wheat; *QRp.slu.4BL* exhibited antibiosis resistance to *R. padi*, while *QRp.slu.5AL* and *QRp.slu.5BL* exhibited tolerance to *R. padi*. In the same study, QTL *QGb.slu*-*2DL* located on chromosome 2DL was shown to be associated with *S. graminum* resistance (Crespo-Herrera et al., 2014). More recently, continuing advances in genome-wide association (GWAS) studies have accelerated the pace of the identification of significant markers or QTLs in aphid resistance genes (Joukhadar et al., 2013).

Taken together, the above findings suggest that true resistance genes to grain aphids were naturally found in wheat gene pools, either by introduction, closely related species or coevolution. Notable examples of aphid resistance genes bred into wheat cultivars resistant to *D. noxia*, for instance *Dn4* derived from wheat line PI 372129, was transferred into several cultivars by adopting cross and backcross techniques, resulting in the release of new wheat cultivars, including “Halt,” “Prowers 99,” “Prairie Red,” and “Yumar” (Smith and Chuang, 2014). Unexpectedly, the transfer of other candidate genes associated with resistance to grain aphids into elite bread wheat lines to construct high-quality wheat germplasm has been relatively unsuccessful.

Similar to chemical control, the practice of breeding for high levels of antibiosis resistance often promotes the development of aphid virulence (reviews by Dogimont et al., 2010; Smith and Chuang, 2014). Additionally, many of the characterized aphid-resistant cultivars are resistant to one species of wheat aphid but are susceptible to other species of aphids (Zhu et al., 2005; reviews by Aradottir and Crespo-Herrera, 2021). For instance, the *T. monococcum* line REB81044 (TM44) is highly resistant to *S. avenae* but susceptible to *R. padi* and *Metopolophium dirhodum* Walker (Tanguy and Dedryver, 2009). These results strongly suggest the need to identify new and diverse aphid resistance genes and genes that confer tolerance or more moderate levels of antibiosis resistance in aphid management, which could be an important hallmark of building plant resistance to aphids, especially in combination with ecological control.

## Herbivore-Mediated Induced Defenses in Plants in Response to Aphid Feeding

**FIGURE 2:**
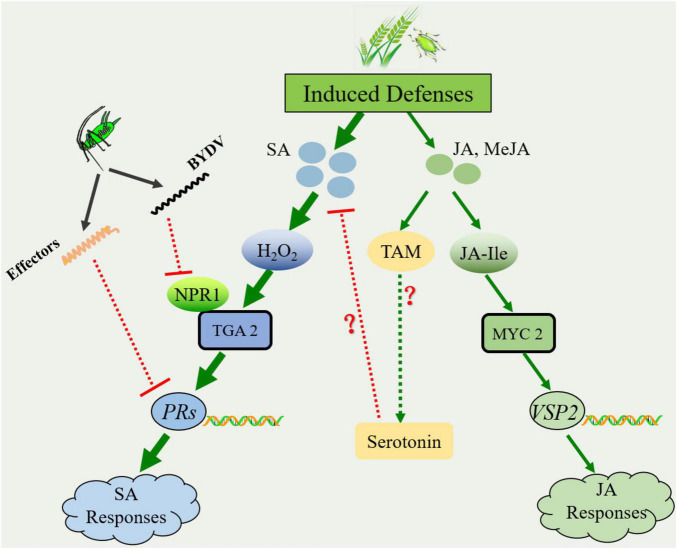
Schematic of the *Sitobion avenae*-wheat interaction during infestation. The colonization of *S. avenae* induces the accumulation of phytohormone molecules, including salicylic acid (SA) and jasmonic acid (JA). SA-mediated defense signaling plays a dominant role in plant defense against subsequent attackers. To diminish SA-dependent responses, JA may promote the synthesis of serotonin. In addition, *S. avenae* could release effectors, and the plant virus carried by the aphids could diminish the host immune response as well. The solid arrow lines represent the pathways supported by experimental evidence from the literature, while the dotted arrow lines represent the pathways predicted from the literature. The red blunt-ends indicate a negative interaction (inhibition) on the SA-mediated plant defense. Red question marks represent the pathways predicted from the literature. H_2_O_2_, hydrogen peroxide; NPR1, non-expressor of pathogenicity-related genes 1; TGA2, transcription factor TGACG binding II; PRs, pathogenicity-related genes; TAM, tryptamine; and VSP2, vegetative storage protein 2.

Cereal plants in agroecosystems are often either sequentially or simultaneously attacked by different species of grain aphids (Ni and Quisenberry, 2006). During feeding and probing, their digestive saliva and honeydew always present a multitude of unknown functions of elicitors derived from the aphid itself or their primary endosymbionts, including EF-Tu, chaperone proteins GroEL, and flagellin, which trigger chemical and morphological responses in attacked plants (War et al., 2012; Sabri et al., 2013; Chaudhary et al., 2014; Jaouannet et al., 2014). Among those plant defense responses, the signaling molecules jasmonic acid (JA) and salicylic acid (SA) play a critical role in mediating the signaling networks involved in the induced defense responses to grain aphids and subsequent conspecific or heterospecific colonizers (Smith and Boyko, 2007). Based on most of the present literature available, JA and its derivatives MeJA are the primary phytohormones in plant defense against chewing insects, while the SA signaling pathway is always involved in defense against piercing-sucking insects (Smith and Boyko, 2007; War et al., 2012). Experimental evidence in sorghum and wheat has suggested that aphid infestation induces rapid and transient emission of SA in host plants (Smith and Boyko, 2007). In seedlings, SA can be perceived and bound by a multitude of SA-binding proteins, including catalase (CAT) and ascorbate peroxidase (APX), resulting in the accumulation of H_2_O_2_ in the apoplastic and symplastic regions of the host (Durner and Klessig, 1995; Tian et al., 2012; Kumar, 2014). H_2_O_2_ could trigger systemic acquired resistance, which often coincides with a programmed cell death (PCD)-type response and a hypersensitive response (HR) that isolates subsequent aphid colonizers and deprives them of nutrients required for subsequent infestation (Johnson et al., 2003; Mou et al., 2003; Tian et al., 2012; Wu et al., 2012). For instance, our latest study suggested that infestation with *R. padi* significantly increased the expression level of the PR-1 gene associated with SA-dependent responses in the resistant winter wheat line 35-E4 (Luo et al., 2020). Meanwhile, increasing experimental evidence has revealed that aphid infestation triggers the expression of genes related to JA and SA synthesis (Figure 2; Zhao et al., 2009; Cao et al., 2014; Luo et al., 2020). For instance, the relative expression of JA synthesis genes, including the *LOX* and *AOS* genes, significantly increased after *R. padi* preinfestation in wheat seedlings of lines 35-E4 and susceptible lines 35-A20 (Luo et al., 2020). The accumulation of JA in wheat seedlings may then be conjugated with the amino acid isoleucine (Ile) to form JA-Ile conjugation with jasmonate-resistant1 (JAR1) (Staswick and Tiryaki, 2004). JA-Ile can be bound by coronatine insensitive 1 (COI1), which promotes the degradation of jasmonate-ZIM domain (JAZ) repressors through the 26S proteasome-mediated pathway (Luo J. et al., 2016). After that, the transcription factor MYC2 in JA signaling was released and positively regulated the transcription of its downstream MYC2-targeted transcription factors to activate JA-induced defense responses, including the expression of the *PDF1.2* (plant defensin 1.2) or *VSP2* (vegetative storage protein 2) genes (Luo J. et al., 2016; Du et al., 2017). However, that study did not determine the expression profiles of marker genes associated with JA-induced defense responses.

Additionally, in many herbivore-plant systems, the interactions between the signaling pathways for SA and JA have been shown to be antagonistic (Shigenaga and Argueso, 2016; Xu et al., 2019; Tan et al., 2021). Over the past decades, a multitude of regulators associated with the antagonistic interaction between SA and JA signaling pathways in plant immune responses have been identified (Pandey et al., 2016; Shigenaga and Argueso, 2016). For instance, MPK4 (mitogen-activated protein kinase 4) positively regulates JA-induced genes such as *PDF1.2* and promotes JA responses while simultaneously suppressing SA biosynthesis and the SA signaling pathway (Petersen et al., 2000; Gao et al., 2008). The central positive regulator of SA signaling, NPR1, can suppress the expression of the genes *PDF1.2* and *VSP2*, markers of the JA signaling pathway (Spoel et al., 2003; Pandey et al., 2016). Additionally, the transcription factor TGA2 acts as an activator of the SA-signaling pathway and as a repressor of JA-responsive genes, probably because TGA2 can bind to the promoter region of ORA59 (octadecanoid-responsive *Arabidopsis* apetala 2/ethylene response factor domain protein 59), which is the master regulator of the JA/ET-induced defense response (Ndamukong et al., 2010; Zander et al., 2014; Pandey et al., 2016). Moreover, herbivore-induced responses in host plants can potentially have a species-specific effect because cultivars generally confer constitutive defense to different species of herbivores at varying levels. For instance, *R. padi* and/or *S. avenae* induced different expression profiles of host JA- and SA-dependent responses in resistant and susceptible winter wheat lines (Luo et al., 2020). Therefore, advances in our understanding of hormone-mediated signaling cascades have laid the foundation for understanding the role of these hormones in wheat resistance to aphids.

Moreover, herbivorous insects can elicit low-molecular-weight salivary proteins, known as effector proteins, and release them into the tissue of the attacked plants during feeding (Figure 2). Although dozens of salivary proteins have been identified in different species of grain aphids, only a small number of candidate effectors have been characterized (Elzinga and Jander, 2013; Jaouannet et al., 2014). The experimental evidence of the identified effectors in other piercing-sucking pests has shown their function in suppressing plant defenses (Elzinga et al., 2014; Xu et al., 2019). For instance, knocking down the salivary effector *Bt56* in *Bemisia tabaci* significantly reduced the transcript level of marker genes involved in SA signaling in *Nicotiana tabacum* while upregulating the transcription of the JA response gene PDF1.2 (Xu et al., 2019). Moreover, the predicted functions of effectors including Mp55 and Mp10 in *Myzus persicae* (Sulzer) were found to suppress plant defenses (Elzinga and Jander, 2013; reviews by Jaouannet et al., 2014). Thus, additional effort is required to study the significance and molecular mechanism of salivary proteins in plant-wheat interactions.

In addition, advances in understanding the interactions between wheat and *Fusarium graminearum* Schwabe (anamorph, Hypocreales: Nectriaceae), an economically important cereal pathogen, provide important clues for understanding the role of JA in the suppression of SA-mediated plant defense during wheat-aphid interactions (Drakulic et al., 2015; De Zutter et al., 2017; reviews by Luo et al., 2021). For instance, *F. graminearum* inoculation leads to an upregulation of candidate genes associated with auxin and serotonin biosynthesis in wheat tissue (Qi et al., 2016; Brauer et al., 2019; Su et al., 2021). Based on the available literature, the accumulation of these two compounds probably occurs because of changes in the JA levels in the environment (Qi et al., 2016; Lu et al., 2018; Yang et al., 2018; Su et al., 2021). The potential role of auxin in wheat–*F. graminearum* interactions revealed that auxin and JA acted synergistically to attenuate the SA-dependent responses. Moreover, the experimental evidence attained from a rice-planthopper system revealed that serotonin could enhance the fitness of planthoppers by establishing a competition between the same precursor chorismite and SA (Lu et al., 2018). However, the underlying molecular mechanism of auxin and serotonin in the suppression of SA signaling remains unknown (Luo et al., 2021). Therefore, the significance of JA in the biosynthesis of serotonin and/or auxin after wheat aphid infestation and its role in enhancing the performance of wheat aphids remain to be investigated.

In response to plant immune cascades, aphids and their transmitted viruses attempt to suppress host plant defenses. For instance, wheat plants infected either by *S. graminum* or *S. avenae* carrying BYDV-GAV significantly reduced the expression level of genes associated with JA- and SA-dependent responses in their hosts, including *LOX*, *AOS*, *NPR1*, and *PAL* genes (Kang et al., 2021). In addition, the viral suppressor of RNAi (VSR) 2b protein of cucumber mosaic virus (CMV), carried by the green peach aphid *Myzus persicae* Sulzer (Hemiptera: Aphididae), contributes to ROS production and directly interacts with the JAZ protein, thereby suppressing JA-responsive genes such as transcription factors MYC2, MYC3, and MYC4 in *Arabidopsis* (Wu et al., 2017; Guo et al., 2019). However, more experimental evidence will be required to confirm the possibility and mechanism by which wheat aphids and their transmitted viruses suppress SA-mediated defense responses in host plants.

Altogether, those regulators and growth-promoting phytohormones triggered by different attackers could fine-tune the plant immune responses, which further aggravates the problem caused by grain aphids in agroecosystems (Figure 2). Therefore, wheat cultivars that incorporate qualified constitutive and induced defenses are preferable for plant breeders to develop novel cultivars with more stable and durable resistance.

## RNA Interference-Based Aphid Control

Since the discovery that double-stranded RNA (dsRNA) can suppress the transcript abundance of target genes, plant- and insect-mediated RNA interference (RNAi) has been developed as a novel potential approach for pest control (Pitino et al., 2011; Xu et al., 2014; Chung et al., 2018; Yang et al., 2019). Over the past decades, plant-mediated RNAi has knocked down the transcript abundance of critical pest genes in numerous herbivore-plant systems, including cotton bollworm-cotton, corn rootworm-maize, planthopper-rice, aphid-tobacco, and aphid-wheat systems, resulting in the disruption of herbivore performance on plants (Pitino et al., 2011; Xu et al., 2014; Yang et al., 2019). For instance, transgenic wheat plants expressing dsRNA of the *carboxylesterase E4* (*CbE E4*) gene fragment of *S. avenae* showed decreased transcript levels of the *CbE E4* gene and impaired herbivore tolerance to phoxim (O,O-diethyl-O-α-oximinophenyl cyanophosphorothioate) insecticides (Xu et al., 2014). Furthermore, rapid advances in wheat genome sequencing and analysis will facilitate the expression of the dsRNA of many target genes involved in the growth, survival or development of grain aphids in transgenic wheat plants.

In addition, dsRNA could be directly delivered via artificial diets or injected into the hemolymph of insects (Pitino et al., 2011). The preference of those two methods depends largely on the size of the herbivores and the skill of the operator. Previous work confirmed that injection is the most widely adopted method to deliver dsRNA molecules into herbivores such as mosquitoes, beetles, honeybees and grasshoppers, and this method achieves more efficient target gene suppression than the dietary method (Xu et al., 2014). In comparison, delivery via an artificial diet is a non-disruptive technique, preserving the integrity of the treated herbivores, but the precise amount of dsRNA taken up cannot be monitored, resulting in low-efficiency suppression (Sapountzis et al., 2014). Although experimental evidence demonstrated successful direct injection of dsRNA of *Ap-crt* and *Ap-cath-L* genes into the salivary glands of the pea aphid *A. pisum* for silencing the salivary gland proteins, in most cases, the delivery of dsRNA into aphids can be achieved following the oral delivery of RNAi in a filter-sterilized liquid diet, similar to plant phloem sap, supplemented with dsRNA. Attempts to transfer dsRNA into pea aphids have successfully knocked down the expression of aphid genes and suppressed their performance, probably because the pea aphid genome sequence is available. Although the sequences of most grain aphid genomes are not available, the accessibility of the wheat and pea aphid genome sequences would provide valuable evidence for constructing dsRNA of crucial genes of grain aphids.

Endosymbionts are harbored by almost all aphids. *Buchnera aphidicola* is the obligate species, that can synthesize missing essential amino acids and B vitamins and improve the nutritional composition of the restricted diet acquired from plant phloem sap (Douglas, 2014). When bacterial symbionts are eliminated from their insect host by antibiotic treatment, the insects grow poorly and produce few or no offspring (Douglas, 2014). It is, therefore, very probable that targeting symbiosis-related insect genes by RNAi in the symbiotic aphid-*Buchnera* system may reduce aphid damage. The *amiD* and *ldcA1* genes present in *A. pisum*, associated with protecting *Buchnera* from host attack, were used as templates, and dsRNA fragments were synthesized for use in liquid artificial diets (Chung et al., 2018). The dsRNA fragments, once distributed within aphids, led to a reduction in the amount of the bacterial symbiont *Buchnera* in the pea aphid, with poor aphid performance (Chung et al., 2018). Taken together, feeding of dsRNA molecules targeting critical aphid genes, either by artificial spraying or specifically expressing them in transgenic plants, may be a promising aphid control approach in the future.

## Conclusion and Future Perspectives

**FIGURE 3:**
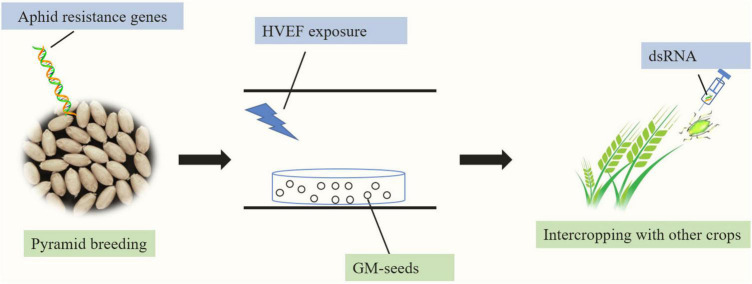
Model summarizing sustainable pest management approaches for cereal aphids in agricultural production. Pyramiding different aphid resistance genes into elite wheat lines to develop aphid-resistant wheat plants and integrating breeding with HVEF exposure of the seeds and intercropping with other crops will be the most promising and effective management strategy for wheat aphid control. The direct transfer of the dsRNA of aphid genes into grain aphids could be a promising aphid control approach.

In the present review, we summarize the present literature on diverse measures known to suppress grain aphid populations. Based on the available data, we propose that the use of aphid-resistant crop plants integrated with agricultural and/or other management practices will be the most promising and effective management strategy for wheat aphid control (Figure 3). In addition, for developing aphid-resistant wheat cultivars, identifying the diverse genes that confer tolerance or more moderate levels of antibiosis resistance is essential for future efforts to improve aphid plant resistance. Moreover, RNAi-mediated aphid control may be an alternative approach for restricting the performance of aphids.

The newly released sequences of common wheat genomes have begun to provide the first real insights into the function and location of grain aphid resistance genes, which will be integrated into elite bread wheat lines to construct high-quality wheat cultivars (Appels et al., 2018). Moreover, the expense and time associated with high-throughput sequencing have been significantly reduced. This will accelerate the process of identifying and utilizing candidate genes with clear molecular mechanisms related to aphid resistance in wheat germplasms. Unfortunately, most of the characterized aphid-resistant cultivars are resistant to one species of wheat aphid but not others. However, wheat aphids are more likely to coinfest different parts of the same plant to obtain nutrients. For example, *S. avenae* prefers to colonize the upper, mature leaves and heads of wheat plants, whereas *R. padi* prefers to colonize the leaf sheaths and the lower leaves (Ni and Quisenberry, 2006). More recently, CRISPR–Cas9 technology has been successfully applied to inactivate crucial genes in cereal crops (Zhang et al., 2016; Kim et al., 2018). Therefore, the combined use of MAS and other molecular breeding measures (pyramiding breeding) is essential for accelerating the breeding of superior cultivars that can withstand attack from different species of grain aphids.

In addition, genetic plasticity not only stimulates grain aphids to evolve insecticide resistance but also serves as the genetic basis for aphids to express virulence to plant genes used in monogenic-based antibiosis resistance. During the plant immune response, the novel feeding effectors secreted by avirulent aphids are sometimes not recognized by the defense system of the resistant plant, and then the virulent aphid overcomes the plant resistance gene or genes in resistant wheat varieties, resulting in outbreaks of grain aphids (Smith and Chuang, 2014). The biotype variation among different RWA isolates and greenbug biotypes supports this conclusion. Historically, more than 11 RWA biotypes and eight greenbug biotypes have been described worldwide (Smith and Chuang, 2014). Although breeding resistant cultivars with multiple, quantitative loci or recessive loci offers a promising approach to delay or avoid aphid virulence, this is a long-term process that can be extremely challenging for plant breeders and entomologists. Altogether, based on the present literature, wheat aphids rapidly evolve virulence to resistant wheat hosts during wheat-aphid interactions, resulting in a need to develop novel strategies for aphid control. Although this method improves the efficiency of downregulation of the expression of grain aphid genes, alternative methods of transferring dsRNA into grain aphids should be explored.

In addition, in agroecosystems, wheat plants are often challenged by different species of pests, including aphid-transmitted viral or other pathogenic diseases, either sequentially or simultaneously. In most cases, there is a “synergistic” relationship between the different species of colonizers. For instance, *S. avenae* pre-exposure significantly facilitates the disease progression of fusarium head blight, a destructive cereal disease. Although the feeding behavior of wheat aphids could trigger hormone-dependent responses in host plants, the role and mechanism of phytopathogen-elicited phytohormones coordinated with other JA or SA signaling pathways to fine tune the plant defense response of wheat remain rudimentary, and further research is required on the crosstalk of complex phytohormonal pathways involved in plant immune responses (Luo et al., 2021). If confirmed, the hormonal crosstalk induced by multiple colonizers would further aggravate the challenge of the ecological regulation of wheat aphid pests in agroecosystems. Therefore, the above working hypothesis triggers important questions for future research and the elucidation of the interaction between aphids and different species of colonizers in ecological regulation of grain aphids and the maintenance of wheat production and grain quality.

## Author Contributions

KL and ZK conceived the study. KL and XW collected the data and led the writing of the manuscript. KL, ZK, XW, and HZ participated in data interpretation and revised the manuscript. KL prepared the figures. All authors have read and approved the manuscript for publication.

## Conflict of Interest

The authors declare that the research was conducted in the absence of any commercial or financial relationships that could be construed as a potential conflict of interest.

## Publisher’s Note

All claims expressed in this article are solely those of the authors and do not necessarily represent those of their affiliated organizations, or those of the publisher, the editors and the reviewers. Any product that may be evaluated in this article, or claim that may be made by its manufacturer, is not guaranteed or endorsed by the publisher.
